# Warwick Edinburgh Mental Well-Being Scale (WEMWBS): measurement invariance across genders and item response theory examination

**DOI:** 10.1186/s40359-022-00720-z

**Published:** 2022-02-18

**Authors:** Joshua Marmara, Daniel Zarate, Jeremy Vassallo, Rhiannon Patten, Vasileios Stavropoulos

**Affiliations:** 1 Institute for Health and Sport, Victoria University, PO Box 14428, Melbourne, 8001 Australia; 2 Department of Psychology, University of Athens, Athens, Greece

**Keywords:** Subjective well-being, Measurement invariance, Item response theory, Psychometric properties, Positive psychology, Gender

## Abstract

**Background:**

The Warwick Edinburgh Mental Well-Being Scale (WEMWBS) is a measure of subjective well-being and assesses eudemonic and hedonic aspects of well-being. However, differential scoring of the WEMWBS across gender and its precision of measurement has not been examined. The present study assesses the psychometric properties of the WEMWBS using measurement invariance (MI) between males and females and item response theory (IRT) analyses.

**Method:**

A community sample of 386 adults from the United States of America (USA), United Kingdom, Ireland, Australia, New Zealand, and Canada were assessed online (N = 394, 54.8% men, 43.1% women, *M*_age_ = 27.48, *SD* = 5.57).

**Results:**

MI analyses observed invariance across males and females at the configural level and metric level but non-invariance at the scalar level. The graded response model (GRM) conducted to observe item properties indicated that all items demonstrated, although variable, sufficient discrimination capacity.

**Conclusions:**

Gender comparisons based on WEMWBS scores should be cautiously interpreted for specific items that demonstrate different scalar scales and similar scores indicate different severity. The items showed increased reliability for latent levels of ∓ 2 *SD* from the mean level of SWB. The WEMWBS may also not perform well for clinically low and high levels of SWB. Including assessments for clinical cases may optimise the use of the WEMWBS.

## Introduction

To date, there has been considerable attention on the existence of diseases and health-related issues as indices of health status, centring mostly on illness and pathology [62]. As posited by Seligman and Csikszentmihalyi, “the exclusive focus on pathology that has dominated so much of our discipline results in a model of the human being lacking the positive features that make life worth living” [64, p. 5]. Over several decades, however, there has been a paradigm shift, where the relevance of individual virtues, strengths, and areas of subjective well-being (SWB) have been proclaimed [41, 47, 62].

Several social scientists and philosophers have concerned themselves with defining happiness or SWB. The construct of SWB has three distinctive features: It is intrinsic within one’s experience; SWB comprises positive measures––it is not merely the absence of negative aspects; SWB measures typically include a holistic assessment of all aspects of a person’s life [13, 22]. However, as satisfaction or affect within life domains may be assessed, the significance is centred on integrated judgment of one’s life [22].

It is not uncommon to see gender comparisons as a focal point in research on distinct psychological characteristics [3]. Gender differences along with the role of gender in SWB has been of much interest [3]. Over several decades, research has shown that men have significantly greater levels of SWB (i.e., [3, 32, 71]). Fewer studies, however, have shown the inverse (i.e., [29]). Further complicating the matter, several studies have found no significant differences in men and women regarding SWB, even after controlling for some demographic factors (i.e., marital status, age, etc.) (i.e., [37, 44, 66, 80, 83]).

Several theoretical approaches could outline why there are such variations in gender differences regarding SWB. Conflicting and inconsistent findings between gender could be attributed to SWB consisting of three dimensions, including life satisfaction, positive affect, and negative affect [3, 21, 24]. The direction and magnitude of gender differences disunite for the separate dimensions, which may have conflation within the analyses, in turn reducing any observed differences [3, 25, 56].

Social construction theorists believe that men may experience poorer SWB than women due to the pressures to adhere to stereotypic beliefs (i.e., [8]). Researchers posit that adherence to masculinity norms in men contributes to harmful social relationships [33, 45] and heightened levels of psychological distress [58]. More specifically, men who endorse the self-reliance norm may value independence and therefore avoid mental health guidance, heightening psychological distress and reducing SWB [38, 49]. Contrarily, women may experience poorer SWB than men due to the power structure in society [55]. Additionally, on average, women are not as financially stable as men, are more likely than men to be sexually harassed within their occupation, feel ‘burn out’ [55].

Various scales have been employed to measure SWB in men and women, including the Positive and Negative Affect Scale [81], the Satisfaction With Life Scale [23], the Scale of Psychological Well-Being [61], and the Short Depression-Happiness Scale [40]. Whilst these scales aim to measure SWB, they do not holistically capture the full conception of SWB, including psychological functioning, cognitive-evaluative dimensions, and affective-emotional aspects. Of late, researchers have consistently used the Warwick-Edinburgh Mental Well-Being Scale (WEMWBS) [1, 72, 75]. The WEMWBS encapsulates a holistic conception of SWB, and it has demonstrated sound psychometric properties exhibiting acceptable validity and reliability [15, 46, 51, 66].

In a series of examinations on the WEMWBS, some studies have shown no significant differences in SWB in men and women [15, 18, 66, 77], however, others have shown that there is a significant difference between men and women regarding SWB [51, 75, 79]. Given these discrepancies, one could assume that the WEMWBS may operate differently between male and female respondents warranting additional investigations into potential issues around differential item functioning across gender groups. While researchers have traditionally employed well established paradigms to assess psychometric properties of an instrument (i.e., classical test theory, CTT), recent technological developments enabled the employment of alternative perspectives. Whilst few studies have explored the psychometric properties of the WEMWBS with newly formed approaches [7], more research is needed to further validate the psychometric properties of the WEMWBS across genders using item response theory (IRT). What’s more, perhaps differences in gender may be due to a lack of understanding and available empirical evidence supporting the robust psychometric properties of this measurement. Establishing the psychometric properties of the WEMWBS can be useful for information interventions, along with assisting clinicians in appraising the impact of one’s services on people’s lives, but also evaluate which aspects of their lives people are displeased with. This will allow clinicians to tailor their services to men and women to meet their needs. To address this aim, the current work will utilise two statistical methods: measurement invariance (MI [59]) and IRT. The following section will identify MI across gender regarding the WEMWBS.

### Measurement invariance (MI)

MI is a statistical method to evaluate whether the psychometric properties of a given measure are stable (i.e., invariant) across groups of interest [8]. For example, one could evaluate whether the WEMWBS assesses SWB in men and women in the same manner. Observing non-invariant responses to WEMWBS items in men and women would indicate that items need to be weighted to obtain similar responses across groups, or that conceptual differences in SWB exist across genders [69]. Specifically, Multigroup Confirmatory Factor Analysis (MCFA) can be employed in the evaluation of MI because it enables structural comparisons at various levels including: configural (i.e., factorial structure); metric (i.e., factor loadings); scalar (i.e., intercepts and thresholds); and strict (i.e., residuals) invariance [31, 54]. In this regard, acquiring configural invariance suggests that the pattern of item-factor loadings along with the number of factors within the WEMWBS are alike for men and women [85]. Moreover, attaining metric invariance for the WEMWBS would suggest that the item-factor loading relationship is being measured with the same metric scale for both groups [69]. Last, confirming scalar invariance for the WEMWBS proposes that the item intercept values are equal across groups. Whilst testing for error/residual variance across groups can be estimated, investigating this layer of invariance is often overlooked [68]. As the residual variance is anticipated to be random, examining their intergroup equality may result in redundant and overly strict models [9].

Tennant and colleagues’ [75] invitation for further investigation of the WEMWBS’ equivalence of psychometric properties across the two genders has been examined in Australian [36], Northern Irish and Scottish [53], Danish [43], and Norwegian [67] samples. Studies evaluating WEMWBS MI across binary gender groups compared goodness-of-fit (GOF) indices (such as comparative fit index, CFI; and root mean standard error or approximation, RMSEA) to determine whether WEMWBS items were indeed invariant [36, 53, 67]. Additionally, bootstrapped likelihood ratio was tested (BLRT [43]) to evaluate MI between gender groups [17]. These studies concluded that gender invariance was consistently observed at the configural and metric levels, and sometimes observed at the scalar level (with non-invariance observed in Australian samples [36]). The sensitive nature of χ^2^ tests to large sample sizes often results in an unnecessarily ‘stringent’ approach, thus differences in GOF indices (i.e., CFI and RMSEA) have been the preferred method to evaluate invariance in SWB across gender groups [9, 36, 53, 67].

### Item response theory (IRT)

IRT is a relatively modern technique that is often projected to overcome some of the limitations that exist with Classical Test Theory (CTT; [18]). First, CTT assumes that the best possible individual score is a composite of observed scores and error resulting in sample-dependent inferences [26]. This results in a major limitation often called sample dependency [27]. Alternatively, IRT emphasises item-person relationships enabling inferences to be made at different levels of the latent trait and thus be sample independent. Second, unlike CTT, IRT can estimate reliability coefficients at the test and item level [27]. Analysing reliability coefficients at the item level can provide greater insights into measurement reliability, enabling a robust evaluation of internal construct and item validity [20].

In the context of IRT, the item-participant relationship is represented by the probability that participants with a certain level of the latent trait (in this case SWB) will endorse a particular item [26]. For example, students with greater math capabilities will be more likely to respond correctly to a difficult math item. This is graphically represented by the item response function (IRF) through a nonlinear (logit) regression line [26]. The exact value of the probability that an individual will endorse an item depends on a set of item parameters including item difficulty (β) and discrimination (α). Difficulty (β) specifies the level of the latent trait required where a participant will endorse a specific item or criterion [31]. For example, ‘easier’ items have lower β values and their IRF is displayed closer to the horizontal axis. In this context, easier items may be endorsed by most participants because it would require little SWB to agree with the proposed criterion/statement. Contrarily, those who endorse ‘difficult’ items may have higher SWB [26]. Discrimination (α) describes how steeply the rate of endorsing an item varies considering the level of the latent trait in each participant [31]. Therefore, items more strongly related to the latent variable present steeper IRF functions and can accurately discriminate different levels of the latent trait (i.e., SWB). IRT models differ according to the estimated number of parameter logistic (PL; [20]). For example, Rasch models behave like 1PL models and assume equal α across different items. Alternatively, Graded Response (GRM) or Generalised Partial Credit (GPCM) models behave like 2PL models and include free estimation of β and α across items [26]. To maximise information attained utilising IRT and seeing as the WEMWBS was measured employing a 5-point Likert scale, the GRM and GPCM were assessed.

Additionally, differential item functioning (DIF) methods can be used to determine whether men and women respond differently to specific items within the WEMWBS [63]. There are three reasons why IRT methods may be more suitable than CTT methods to detect DIF [12]: (i) IRT provides more accurate statistical properties of items than CTT to ascertain where the item functions differently (i.e., difficulty, discrimination, or pseudo-guessing); (ii) item parameter estimates derived from IRT are less confounded and influenced with sample specific characteristics; (iii) finally, the item characteristic curve (ICC) for each group (men and women) can be exhibited via graphic illustration, which increases the comprehensibility of items displaying DIF [12].

### Present study

While WEMWBS psychometric properties have been examined with IRT models, some authors (i.e., ([2, 34, 73]) were limited to Rasch Models to assess item-participant relationships. Additionally, one study investigated the psychometric properties of the WEMWBS between participants under 65 and over 65 years of age employing GRM and GPCM that freely estimate item discrimination (α; slope), and item difficulty (β; location) parameters [57]. To our knowledge, however, no other research has examined the WEMWBS employing GRM and GPCM that freely estimate item discrimination (α; slope), and item difficulty (β; location) parameters in men and women. Subsequently, the present study aims to extend on previous findings related to the psychometric properties of the WEMWBS in two meaningful ways: (a) it aims to expand gender MI findings using relaxed research methods (i.e., ΔCFI, ΔRMSEA) from a different national sample; and (b) it will be the first to investigate the DIF of the WEMWBS items through GRM and GPCM models for participants with differing levels of SWB. This is noteworthy in three ways. Firstly, it will add clarity regarding the comparability of men and women from scores within the WEMWBS in both clinical practice and research. Secondly, it will allow ranking of the WEMWBS items based on their psychometric performance (i.e., item priority ranking). Finally, it will inform how particular items from the WEMWBS may provide reliable and/or less reliable information among men and women with both higher and lower levels of SWB. We expect the scale to be invariant across gender and to have differing levels of reliability across different responses and scale scores.

## Method

### Participants

Upon receiving approval from the Victoria University Ethics Committee, participants were recruited online via a crowd sourcing platform (Prolific.co) and were awarded $2.50 each for their time. As part of a larger study, 394 participants completed an online survey including the WEMWBS. Omission of items was not allowed by the Qualtrics-setting parameters. These included 216 men and 170 women, whilst eight participants identified as non-binary. These eight participants were excluded in the present analyses targeting gender differences. The remaining participants’ age ranged from 18 to 39 years (*M* = 27.54, SD = 5.58). Only the 386 full responses were utilised for statistical analyses resulting to a maximum random sampling error of 0.089 for a 95% confidence interval and 0.117 for a 99% confidence interval. Most participants were heterosexual (80.5%), had an undergraduate degree (40.4%), worked full-time (44.3%), lived in the United States of America (USA; 54.9%), and reported Caucasian ethnicity (57.8%).

### Measures

The WEMWBS is a 14-item scale; each answered on a 1 to 5 Likert scale, ranging from *“none of the time”* to *“all the time”*. Items cover different aspects of eudaimonic and hedonic well-being and are worded positively such as *“I’ve been feeling relaxed”*, and *“I’ve been dealing with problems well”* [75]. The overall score is calculated by summing the scores for each item, with the minimum overall score being 14 and maximum score being 70. A higher score indicates a higher level of SWB [75]. Table 1 presents a description of the items and descriptive statistics for the current sample. Previous research found a unidimensional factor structure, along with strong internal consistency (Cronbach’s α = 0.91), construct validity and test–retest reliability (r = 0.83) in student samples of men and women [75]. Additionally, the internal consistency of the WEMWBS in the present study was acceptable (Cronbach’s α = 0.94, McDonald’s ω = 0.95).

**Table 1:** Descriptive statistics for WEMWBS 14 items (N = 386)

	Overall				Men	Women
	*M*	*SD*	Skewness	Kurtosis	*M*	*M*
1. I’ve been feeling optimistic about the future	3.04	1.04	− 0.30	− 0.49	3.05	3.02
2. I’ve been feeling useful	3.07	0.99	− 0.15	− 0.52	3.06	3.08
3. I’ve been feeling relaxed	2.97	0.90	− 0.01	− 0.59	3.07	2.85
4. I’ve been feeling interested in other people	3.12	1.03	− 0.26	− 0.63	3.13	3.12
5. I’ve had energy to spare	2.65	1.05	0.19	− 0.53	2.97	2.38
6. I’ve been dealing with problems	3.03	0.96	− 0.07	− 0.28	3.10	2.95
7. I’ve been thinking clearly	3.31	0.96	− 0.33	− 0.17	3.38	3.22
8. I’ve been feeling good about myself	2.98	1.05	− 0.02	− 0.53	3.07	2.87
9. I’ve been feeling close to other people	2.98	1.11	− 0.10	− 0.76	2.97	3.00
10. I’ve been feeling confident	2.93	1.21	− 0.04	− 0.69	3.08	2.74
11. I’ve been able to make up my own mind about things	3.39	1.03	− 0.41	− 0.19	3.40	3.39
12. I’ve been feeling loved	3.26	1.17	− 0.22	− 0.79	3.20	3.34
13. I’ve been interested in new things	3.19	1.08	− 0.23	− 52	3.26	3.10
14. I’ve been feeling cheerful	3.03	1.00	− 0.19	− 0.45	3.06	2.99

*M* mean, *SD* standard deviation

### Statistical analysis

**Fig. 1:**
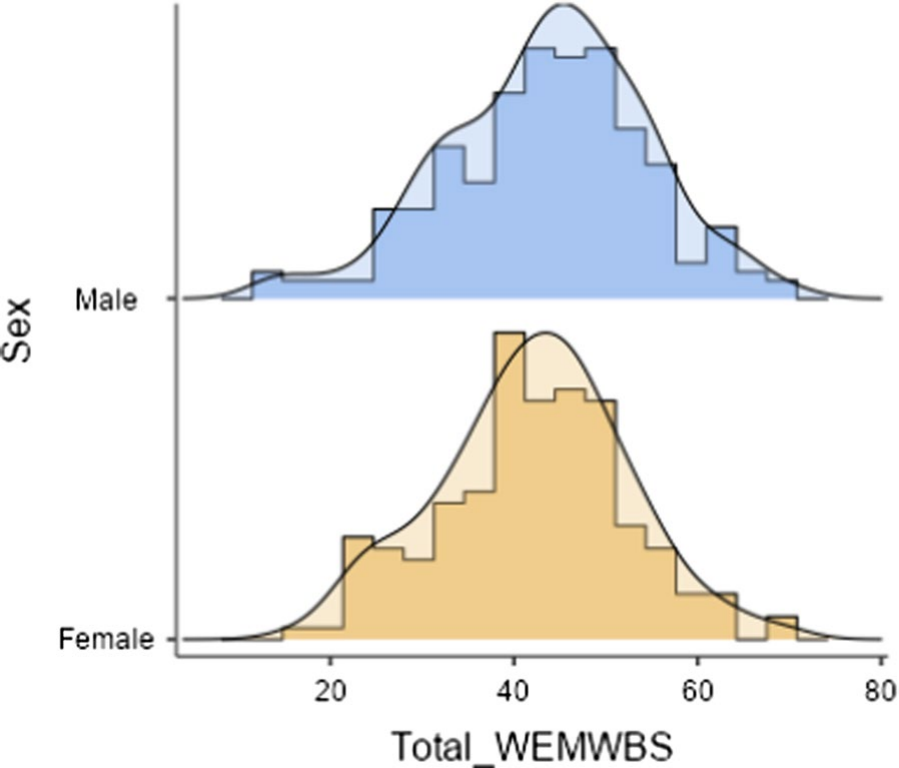
Normality across gender

To address the outlined aims, two statistical analyses were employed: (i) multigroup Confirmatory Factor Analyses (MCFA) to observe MI across men and women and (ii) psychometric examination of the WEMWBS via IRT (including DIF). The Lavaan package [60] in R Studio was employed to conduct tests of MI, and IRTPRO 5.0 was employed to conduct IRT and DIF analyses. Examinations of the skewness and kurtosis across the variables were used to evaluate the assumption of univariate normality (see Table 1). The distributions of all items yielded skewness statistics of less than absolute 3, and kurtosis statistics of less than absolute 8 [28, 42]. Additionally, normality was met across gender (see Fig. 1). Therefore, the assumption of univariate normality was met for this study.

### Groups confirmatory analysis (MCFA) analysis

Incremental fit indices compare the χ^2^ value of a baseline model to that of χ^2^ value in its raw form. Within this group contains the Comparative Fit Index (CFI) and Tucker-Lewis Index (TLI). The CFI analyses the fit of the model by investigating the difference between the hypothesised model and the data [30]. Values of ≥ 0.90 indicate good fit [35]. The TLI measures a relative reduction in misfit per degree of freedom. It is also argued that values < 0.90 indicate a need to respecify the model [35, 52].

Absolute fit indices establish how well a priori model fits the sample data [65] and validates how valid the model fit is. Common absolute fit indices include the standardised root mean square residual (SRMR), and the root mean square error of approximation (RMSEA). The RMSEA and SRMR are the preferred measures for calculating model fit. Values below 0.08 (RMSEA) and under 0.11 (SRMR) are indicative of acceptable fit [35, 74, 76]. At this point it should be noted that the choice of RMSEA and SRMR as appropriate fit indices in the present study aligns with the work of Taasoobshirazi and Wang [74], advocating their use with models involving higher degrees of freedom and bigger sample sizes (N > 100), as is the case here.

First, multiple Groups Confirmatory Analysis (MCFA) analysis was conducted on scales and groups (men and women) [68]. This process involves a stepwise model comparison with progressively restrictive parameters to test for ill-fitting models and subsequently observe sources of non-invariance [9]. In this context, we first compared the configural and metric models. If the test was not significant, metric invariance was established and therefore scalar invariance was tested. Testing scalar invariance requires a similar approach via comparing scalar model against the metric model. If this test was not statistically significant, then this would indicate scalar invariance of the factorial model. Scalar invariance must hold to be able to interpret correlations and latent means across groups (men and women) [78]. If scalar invariance was not met, then adjusting factor loadings and/or intercepts to obtain partial MI could be established [70]. Finally, if either partial or full scalar variance holds, then testing strict invariance by comparing the strict model with the scalar model could be employed. With the lack of strict invariance, groups (men and women) can still be compared on the latent construct (WEMWBS) [78].

Considering that χ^2^ is sensitive to samples over 200 observations, we evaluated differences (Δ) in CFI and RMSEA to test for significant differences between comparison and nested models [9, 68]. Sources of non-invariance were deemed present if any of the following conditions was met: ΔCFI > 0.010, ΔRMSEA > 0.015 [31, 59]. Whilst there are several fit indices, the CFI and RMSEA are the most preferred indices to test invariance, as they are the most regularly reported indices in everyday research [19, 39]. For testing invariance, a change of ≥ 0.010 in CFI, complemented by a change of ≥ 0.015 in RMSEA would indicate non-invariance [16]. All fit indices are shown in Table 2, however, only change (Δ) in CFI and RMSEA are reported. Modification indices were evaluated to determine sources of non-invariance if significant differences between models were observed [9]. Thus, to achieve partial invariance, the highest contributions towards sources of non-invariance were made free in the model until non-significant changes were observed.

**Table 2:** WEMBWS across men and women (N = 386)

	χ^2^	df	*p*	CFI	ΔCFI	TLI	RMSEA	Δ RMSEA	SRMR	BIC	AIC
Configural: loadings + intercepts free	399.266	154	0.233	0.924		0.910	0.091		0.049	13,076	13,076
Metric: loadings fixed + intercepts free	415.117	167	0.001*	0.923	0.001	0.916	0.088	0.003	0.061	13,014	12,734
Scalar: loadings + intercept fixed	476.726	180	0.064	0.908	0.015	0.907	0.092	0.004	0.067	12,999	12,769
Partial invariance	438.214	179	0.001*	0.915	0.008	0.907	0.092	0.000	0.065	13,043	12,758

* = Statistically significant *p* < 0.05. Partial invariance achieved by freeing intercept 5. The model is regarded as acceptable if the chi-square is not significant. However, this is disregarded when the sample size exceeds 200. The Comparative Fit Index (CFI) compares the examined model of interest with the null model. The Tucker Lewis Index (TLI) is computed by the division of the chi square for the target model and the null model by their corresponding df vales (relative chi squares), which are then subtracted from each other, and their difference is finally divided by the relative chi square for the null model minus 1. The Root Mean Square Error of Approximation (RMSEA) represents the square root of the average or mean of the covariance residuals. The Bayesian Information Criterion (BIC) expresses the log of a Bayes factor of the target model compared to the saturated model. Finally, the Akaike information criterion (AIC) is regarded as an information theory goodness of fit measure applicable when maximum likelihood estimation is used [5]. After freeing the intercept for one item (Item 5; *“I’ve had energy to spare”*), partial scalar invariance was supported

### Item response theory (IRT) analysis

Second, WEMWBS psychometric properties were examined using IRT analysis. Local independence and unidimensionality assumptions were assessed prior to the analysis. Local independence assumes that item scores do not correlate when holding the latent trait constant. This is determined by residual correlations on items < 0.1 [20]. Using a CFA analysis, unidimensionality assumes correlations on items are assigned to one factor.

The current study considered three IRT models: (1) partial credit model (PCM), which assumes equal discrimination parameter across items; (2) generalised partial credit model (GPCM), which is flexible with categorical (classes) and linear latent traits; and (3) graded response model (GRM), which compares highest fit models (polytomous) to examine variations (α) using χ^2loglikelihood^ [11, 26].

Whilst IRT models were initially established to evaluate dichotomous data (i.e., yes/no), extensions of these models have been employed to accommodate the use of ordered polytomous data (i.e., more than two response options reflecting order/ranking [26, 85]). Given that the WEMWBS measures SWB with a 5-point scale (with multiple and incrementally ordered answers per item), the use of IRT models suitable for polytomous data is required [85]. In that framework, “Rasch” models assume equal discrimination (α) across items and behave as 1PL models [20]. Contrarily, the generalised partial credit (GPC) and graded response models (GRM) assume variable item discrimination properties (α), and present more suitable for ordered polytomous data [26, 85].

Following past recommendations, we employed marginal likelihood information statistics (M_2_) to assess goodness of fit [10, 11]. However, given that M_2_ is sensitive to samples > 200, RMSEA was emphasised to assess goodness of fit [48]. Additionally, to determine optimal model fit (i.e., GRM vs. GPCM), we considered: (1) the loglikehood index of fit [20]; (2) the Bayesian Information Criterion (BIC); (3) the RMSEA; and (4) the Akaike Information Criterion (AIC), with lower values indicating improved fit [20, 35]. Visual examination was then conducted by the item information function (IIF; [10]) reliability and on Item Characteristic Curves (ICC; α, β). Test Information Function (TIF) and the Test Characteristic Curve (TCC; [10]) was used to assess the test reliability at the scale level. Due to the raw-scale and trait scores, the TCC determined cut-off points determined by two standard deviations (SD) above the mean [26].

## Results

### Confirmatory factor analysis

First, CFA was performed to verify the unidimensionality of the scale. The default estimator in the Lavaan package (maximum likelihood [ML]) was used due to the continuous and normative nature of the data [60]. As investigated by a CFA, the WEMWBS demonstrated acceptable fit (χ^2^ = 334.42, df = 77, *p* < 0.001, CFI = 0.920, TLI = 0.906, RMSEA = 0.093, SRMR = 0.045). It should be noted, however, that the current factorial structure did not attain optimal RMSEA values (i.e., < 0.08; [74]) Nevertheless, we note that recent work of Maydeu-Olivares et al. (2018) [49] suggested that in larger models (*p* ≥ 30), RMSEA is likely to support models as closely not fit, specifically if sample size is less than 500 (as is the case here).

### Measurement invariance

**Fig. 2:**
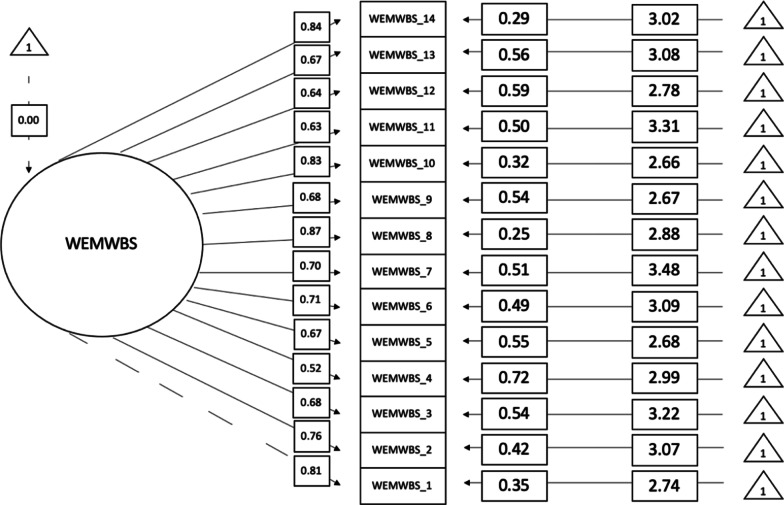
WEMWBS unstandardised item loadings for men. This graph demonstrates the unidimensional factorial structure of the WEMWBS for men. *Note*: WEMWBS_1 = Item 1, WEMWBS_2 = Item 2, etc.

**Fig. 3:**
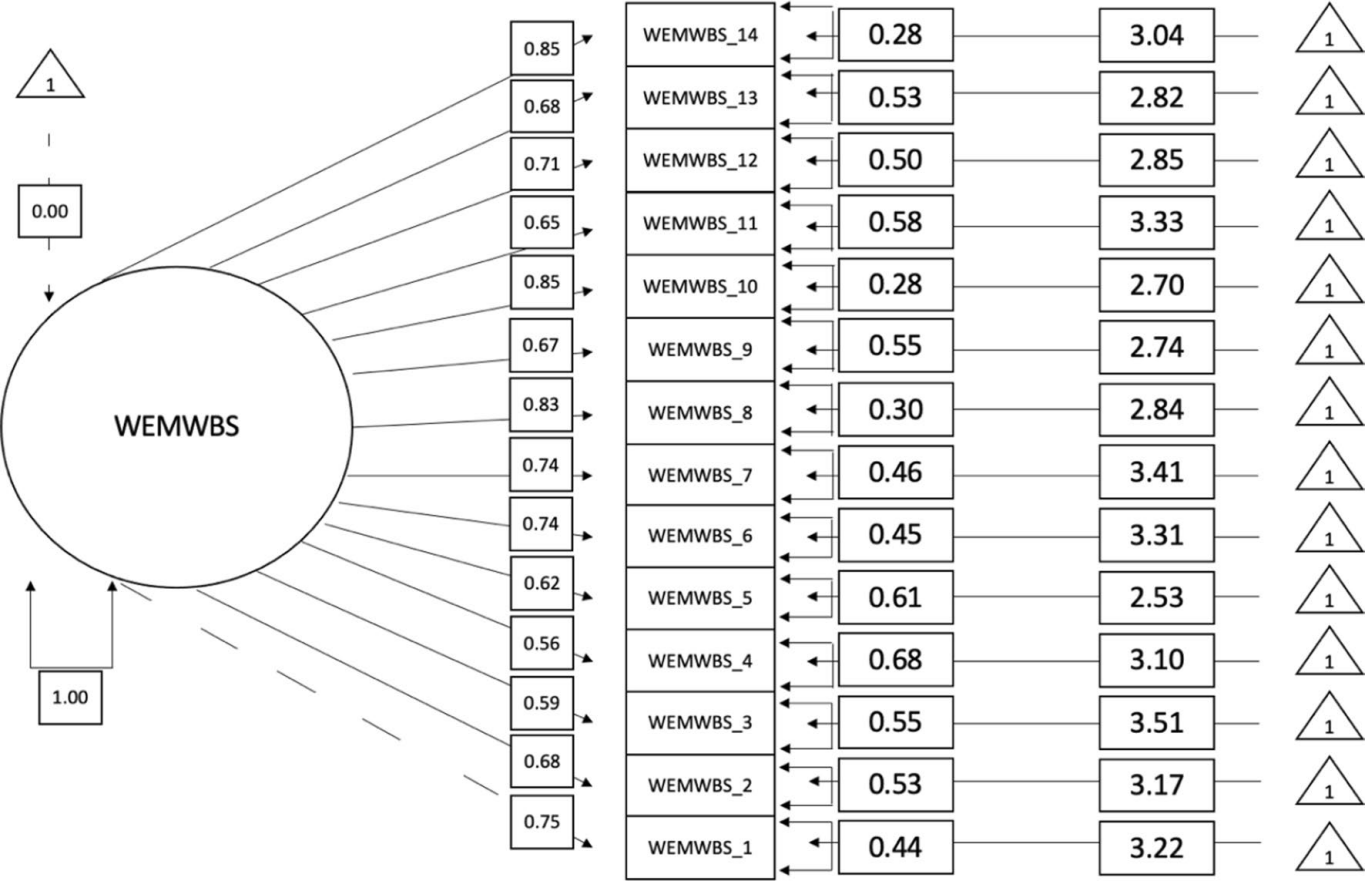
WEMWBS unstandardised item loadings for Women. This graph demonstrates the unidimensional factorial structure of the WEMWBS for women. *Note*: WEMWBS_1 = Item 1, WEMWBS_2 = Item 2, etc.

Second, the WEMWBS unidimensional factorial structure across binary gender groups was assessed. Both groups demonstrated acceptable fit according to acceptance criteria for RMSEA, TLI and CFI [35] (men: χ^2^ = 222.521, *df* = 77, *p* < 0.001, CFI = 0.921, TLI = 0.907, RMSEA = 0.094, SRMR = 0.048) (women: χ^2^ = 176.742, *df* = 77, *p* < 0.001, CFI = 0.928, TLI = 0.915, RMSEA = 0.087, SRMR = 0.049). All loadings were above 0.268 for men (see Fig. 2) and above 0.317 for women (see Fig. 3). The internal consistency of the WEMWBS in the present study was acceptable for men (Cronbach’s α = 0.94, McDonald’s ω = 0.95) and women (Cronbach’s α = 0.93, McDonald’s ω = 0.95).

Third, the unconstrained (both loadings and intercepts free) multi group model was computed and showed good fit (χ^2^ = 399.266, CFI = 0.924, TLI = 0.910, RMSEA = 0.091, SRMR = 0.049). In the next step, metric invariance (fixed loadings and free intercepts) was tested and showed non-significant changes in CFI (∆CFI = 0.001; see Table 2). Scalar invariance (loadings and intercepts free) indicated a significant drop in CFI (∆CFI = 0.015) but non-significant changes in RMSEA (∆RMSEA = 0.004). Partial invariance was tested by freeing each item one-by-one from the nested model and compared to the original model to assess each item’s individual influence on the changes in CFI. This resulted in item 5 as the highest contributor to ∆CFI in the model (∆χ^2^ = 6.718). Thus, item 5 intercept was then configured to be free (relaxed), resulting in a final partial invariance model which had a non-significant change in CFI from the configural and metric model. No further items were relaxed as the strictest model possible is the least complex.

### Psychometric IRT properties

The GRM estimation (M_2_[1442] = 2324.61, *p* < 0.001; χ^2Loglikelihood^ = 12,282.70; RMSEA = 0.04; BIC = 12,699.61; AIC = 12,422.70) showed better fit compared to the GPC model (M_2_[1442] = 4224.35, *p* < 0.001; χ^2Loglikelihood^ = 12,352.72; RMSEA = 0.07; BIC = 12,769.63; AIC = 12,492.72). Similarly, the GRM showed better fit to data compared to the PCM (M_2_[1455] = 2658.76, *p* < 0.001; χ^2Loglikelihood^ = 12,465.62; RMSEA = 0.05; BIC = 12,579.62; AIC = 12,805.10) Discrimination parameters for all items ranged between the moderate and the very high range (0 = non discriminative; 0.01–0.34 = very low; 0.35–0.64 = low; 0.65–1.34 = moderate; 1.35–1.69 = high; > 1.70 = very high; [4]) between 1.29 (α item 4) and 3.83 (α item 8). Similarly, factor loadings ranged in the high range between item 11 and 12 (λ = 0.70) and item 8 (λ = 0.91; [31]). The descending sequence of the items’ discrimination power and loadings is 8, 14, 10, 1, 2, 6, 7, 9, 13, 3, 12, 5, 11, and 4 (see Table 3).

**Table 3:** Item discrimination, difficulty, and loadings of the WEMWBS (N = 386)

Item	Label	*a*	*b*_1_	*b*_2_	*b*_3_	*b*_4_	*Spread*	*λ loadings*
1	WEMWBS_1	2.55	− 1.53	− 0.63	0.47	1.94	3.47	0.83
2	WEMWBS_2	2.29	− 2.01	− 0.68	0.43	2.02	4.03	0.80
3	WEMWBS_3	1.76	− 2.61	− 0.63	0.74	2.77	5.38	0.72
4	WEMWBS_4	1.29	− 2.57	− 0.89	0.39	2.58	5.15	0.60
5	WEMWBS_5	1.69	− 1.45	− 0.13	1.15	2.54	3.99	0.71
6	WEMWBS_6	2.28	− 2.01	− 0.69	0.61	1.98	3.99	0.80
7	WEMWBS_7	2.17	− 2.25	− 1.09	0.17	1.73	3.98	0.79
8	WEMWBS_8	3.83	− 1.44	− 0.45	0.52	1.58	3.02	0.91
9	WEMWBS_9	1.87	− 1.73	− 0.52	0.51	1.97	3.70	0.74
10	WEMWBS10	3.28	− 1.31	− 0.38	0.54	1.60	3.91	0.89
11	WEMWBS11	1.68	− 2.35	− 1.26	0.07	1.61	3.96	0.70
12	WEMWBS12	1.69	− 2.05	− 0.82	0.20	1.41	3.46	0.70
13	WEMWBS13	1.83	− 1.98	− 0.87	0.34	1.72	3.70	0.73
14	WEMWBS14	3.56	− 1.54	− 0.55	0.47	1.81	3.35	0.90

*α* defines the capacity of an item to discriminate between varying levels of SWB (θ). *β* defines the level of behaviour intensity, where subsequent response rates are more probable than their previous rate. Spread is the range of difficulty parameters across the different Likert points. λ defines the amount of variance of an item explained by the latent factor

**Fig. 4:**
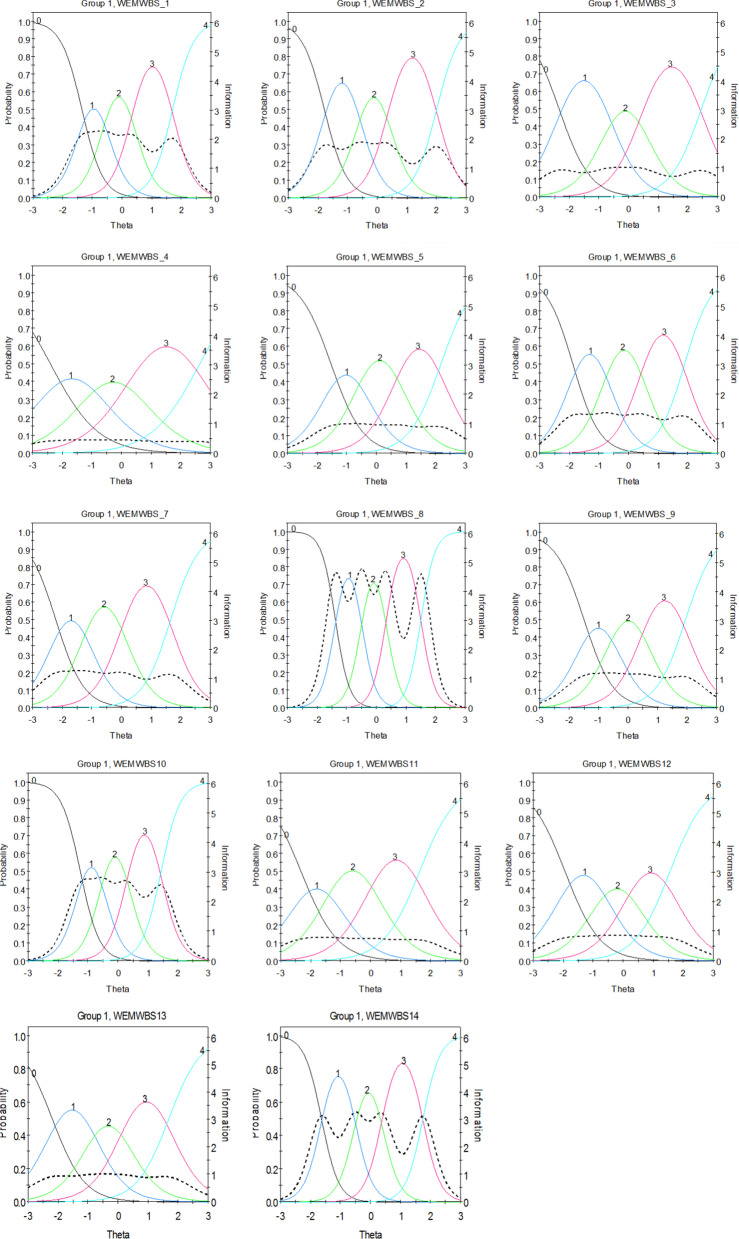
WEMWBS Item Characteristic Curves (ICC) and Item Information Function (IIF) (*N* = *386*). ICC plots demonstrate how the probability of endorsing a category of WEMWBS items (i.e., *none of the time to all of the time*) change as levels of the latent trait change. IIF plots demonstrate how reliability indices vary with changes in the latent trait. *Note*: ICC expresses in a nonlinear (logit) regression line pertaining to difficulty (β) and discrimination (α) parameters. Difficulty (β) indicates the level of the latent trait where there is a .5 probability that a participant will endorse a specific criterion or item [85]. For example, ‘easier’ items have lower β values and their ICC is represented closer to the horizontal axis. For clarification purposes, those endorsing easier items are said to have lower SWB. Conversely, those who endorse the difficult items are said to have higher SWB ([26, 85]). Discrimination (α) describes how steeply the rate of success (positive response) of an individual varies according to their latent trait levels. Thus, items more strongly related to the latent variable present steeper ICC functions [85]

Item difficulty parameters (β) can be observed via ICCs to evaluate how the probabilities of endorsing a category (i.e., *‘always’*) in WEMWBS items change as levels of the latent trait change (Fig. 4). Specifically, ICCs are expressed in a nonlinear (logit) regression to demonstrate potential fluctuations between the different thresholds across items. For example, ‘easier’ items have lower β values and their ICC is represented closer to the horizontal axis. For clarification purposes, those endorsing easier items are said to have lower SWB. Conversely, those who endorse the difficult items are said to have higher SWB ([26, 85]).

Indicatively, for the first threshold the ascending item sequence of difficulty was 1, 10, 8, 5, 14, 9, 13, 2, 6, 12, 7, 11, 4 and 3. Considering the fourth threshold, this alternated to 1, 12, 8, 10, 11, 13, 7, 14, 9, 6, 2, 5, 4 and 3. This is important given the clinical implications of the scale. Understanding the difficulty parameters will allow clinicians to comprehend which items are more difficult for clients to answer and thus require greater levels of SWB, and which items are easier for clients to answer and thus require lower levels of SWB. Nonetheless, the threshold difficulty parameters gradually increased between the first and the last threshold across all items (see Table 3). In sum, IRT investigates showed that: (i) as increasing item scores correctly described increasing levels of SWB behaviours across all items, the rate of these increases differs from item to item, and (ii) different thresholds perform differently from item to item considering their level of difficulty.

Considering the items’ reliability across the different levels of the latent trait, controlling concurrently for the different levels of items’ difficulty, meaningful variations were confirmed. Indicatively, the IIF of items 8, 10 and 14 provided the highest levels of information/reliability, although with some variability (within one standard deviation), in the range between 2 SDs above and below the mean. The IIFs of items 1, 2, 6, 9 and 13 showed rather undifferentiated better performance in the range between 2 SDs above and below the mean with significant drops in the areas of 3 SDs above and below the mean. Items 7, 11 and 12 showed a rather low and undifferentiated level of reliability in the area between minus 3 SDs below the mean and 2 SDs above the mean with a significant drop for behaviours exceeding 2 SDs above the mean. Items 3 and 4 showed undifferentiated low reliability across all the range between 3 SDs below the mean and 3 SDs above the mean. Finally, item 5 showed average reliability for the area between 3 SDs below the mean and up to 2 SDs above the mean and mild to moderate drop for scores around 3 SDs higher than the mean (see Fig. 3).

**Fig. 5:**
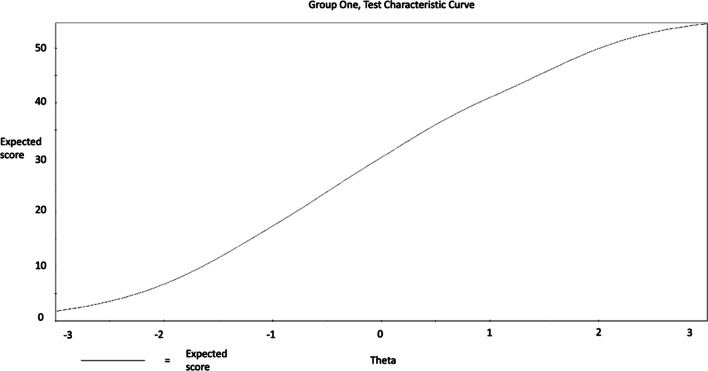
WEMWBS Test Characteristic Curve (TCC) provides a visual representation of expected WEMWBS scores as a function of latent trait levels (i.e., as WEMWBS scores increase, levels of the latent trait increase) for men

**Fig. 6:**
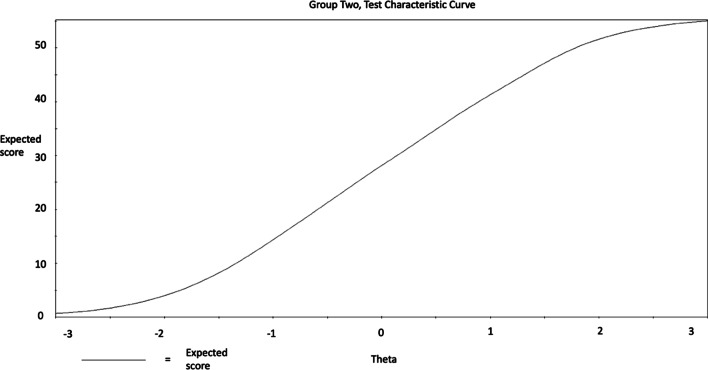
WEMWBS Test Characteristic Curve (TCC) provides a visual representation of expected WEMWBS scores as a function of latent trait levels (i.e., as WEMWBS scores increase, levels of the latent trait increase) for women

**Fig. 7:**
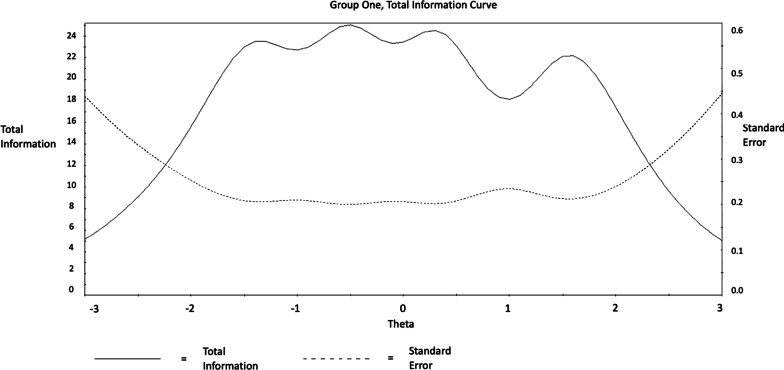
WEMWBS Test Information Function (TIF). Demonstrates the relationship between standard errors and reliability indices (i.e., smaller standard errors result in more information) for men

**Fig. 8:**
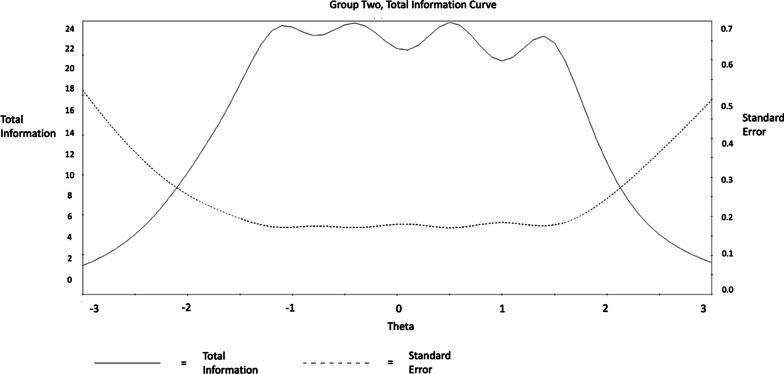
WEMWBS Test Information Function (TIF). demonstrates the relationship between standard errors and reliability indices (i.e., smaller standard errors result in more information) for women

Considering the performance of the scale as whole, this is visualized by the Test Characteristic Curve (TCC) and the Test Information Function (TIF) figures following. The TCC graph illustrates that the trait of SWB inclined steeply, as the total score reported increased (from 10 to 50; see Figs. 5, 6, 7, 8) among men and women. Considering the information provided by the scale, improved information (TIF) scores were around − 1.5 SDs below the mean, up to about + 2 SDs above the mean (see Figs. 5, 6, 7, 8) among men and women.

These findings suggest that the WEMBWS provides a sufficient and reliable psychometric measure for assessing individuals with high and low levels of the mental SWB in the range between 1.5 SDs below and 2 SDs above the mean. Nevertheless, it may not be an ideal measure for individuals with extremely low, or high SWB behaviours in the areas exceeding 2 SDs above or below the mean. SWB at the levels of 2 SDs below and above the mean trait level correspond with raw scores of 7 and 49 respectively, and based on these, it could be suggested as conditional (before clinical assessment confirmation) diagnostic cut-off points [31]. Accordingly, 0% of the participants scored below 2 SD and 22.8% scored above 2 SD and thus were at risk for presenting SWB in the problematic range.

Considering DIF of WEBWBS across men and women, sources of non-invariance at the item level were detected. DIF statistics were observed (see Table 4) for all items, with significant discrepancies across groups (total χ^2^ *p* < 0.05) in item 5. We then anchored invariant items and re-calculated DIF statistics only with non-invariant items (i.e., item 5) to avoid incurring in familywise type I error [85]. Upon anchoring all invariant items, item 5 showed a significant difference in total χ^2^ (*p* < 0.001) and difficulty (χ^2^_cja_ *p* < 0.001). This difference between men and women is seen in Fig. 4, where men exhibit higher probability of endorsing ‘harder’ categories in item 5 (i.e., *“some of the time”, “often”,* and *“all of the time”*); this suggests that it is ‘easier’ for men to score higher in this item.

**Table 4:** Differential item functioning (DIF) statistics for graded items (N = 386)

Item numbers in:										
Group 1 (Men)	Group 2 (Women)	Total *X*^2^	*df*	*p*	*X*^2^_a_	*df*	*p*	*X*^2^_c|a_	*df*	*p*
1	1	5.8	5	0.3245	0.3	1	0.5767	5.5	4	0.2396
2	2	6.0	5	0.3077	0.9	1	0.3442	5.1	4	0.2783
3	3	7.3	5	0.1961	0.4	1	0.5370	7.0	4	0.1378
4	4	5.5	5	0.3551	2.2	1	0.1416	3.4	4	0.4993
5	5	24.7	5	0.0002	0.1	1	0.8010	24.7	4	0.0001
6	6	5.1	5	0.4035	1.0	1	0.3260	4.1	4	0.3879
7	7	3.1	5	0.6832	1.2	1	0.2712	1.9	4	0.7548
8	8	8.3	5	0.1393	0.5	1	0.4607	7.8	4	0.1001
9	9	7.9	5	0.1603	0.1	1	0.7383	7.8	4	0.0986
10	10	11.2	5	0.0482	1.7	1	0.1982	9.5	4	0.0497
11	11	2.1	5	0.8329	1.4	1	0.2457	0.8	4	0.9431
12	12	9.0	5	0.1101	2.6	1	0.1090	6.4	4	0.1719
13	13	8.8	5	0.1185	0.5	1	0.4741	8.3	4	0.0826
14	14	6.8	5	0.2343	1.1	1	0.2918	5.7	4	0.2220

## Discussion

The present study is the first of this type to combine classical test theory and item response theory procedures to assess the psychometric properties of the Warwick Edinburgh Mental Well-Being Scale at both the scale and the item level for an English-speaking sample.

Regarding MI, the loadings and intercepts of item 5 were shown to be non-invariant across men and women, when CFI and RMSEA comparisons were applied. Regarding the IRT evaluation, although all items presented with high discrimination capacity, this fluctuated according to the following descending sequence of items 8, 14, 10, 1, 2, 6, 7, 9, 13, 3, 12, 5, 11, and 4. Similarly, items’ difficulty parameters differed across the different item thresholds. Finally, in relation to the scale, although this seems to perform sufficiently and reliably for examining SWB levels between 2 SDs below and above the mean, this measure of SWB may not be ideal for individuals experiencing extremely low or high SWB (scores that lie ∓ 3 SD beyond the mean).

### Uni-dimensionality and measurement invariance across genders

In conjunction with contemporary research, the WEMWBS demonstrated a favourable unidimensional factorial structure, as all items loaded significantly and saliently on a single latent construct [36, 43, 53, 67]. Furthermore, when dividing the sample into men and women, WEMWBS preserved a suitable unidimensional factorial structure as all items loaded significantly and had an acceptable model fit for both groups. Moreover, when utilising a ‘relaxed’ approach (i.e., changes in CFI and RMSEA [8]) to establish invariance across gender groups, WEMWBS established support for invariance at configural and metric levels, however, non-invariance was observed at the scalar level. Therefore, it could be argued that even though SWB is perceived in the same vein across men and women, gender response patterns across the different items should be interpreted cautiously for non-invariant items.

Support for partial invariance that the degree of the relationship between multiple items is equal across men and women. Moreover, support for partial invariance suggested that sources of non-invariance across men and women were also present in item intercepts. Item 5 demonstrated unequal intercepts between men and women (*“I’ve got energy to spare”*). This may suggest that men and women who experience the same level of SWB may provide differing responses for this specific item. The results show women scored lower on this item and aligns with theoretical explanations [55, 82, 84]. These studies suggest power structures in society including being less financially stable and living under the poverty threshold compared to men, experience occupational sexual harassment, feel ‘burn out’, and distress due to caring for family members [55] might lead to women having less energy to spare.

From a biological standpoint, women’s greater vulnerability to having less energy to burn than men can be explained by a dysregulated hypothalamic–pituitary–adrenal (HPA) axis [55, 82]. As women are more likely than men to have a dysregulated HPA response to stress, this may make them more susceptible to utilising energy in response to stress [55, 82]. Additionally, women reporting lower levels of energy to spare than men can be explained by rapid fluctuations in ovarian hormone levels, which are responsible for the regulation of the HPA axis [55, 84]. Consequently, this may cause some women may experience less energy to spare during puberty, menopause, and premenstrual periods. These changes trigger dysregulation of the stress response, making women during these hormonal fluctuations more susceptible to ‘burn out’ [55, 84].

### Scale and item discrimination, difficulty, and reliability

The findings from the IRT analysis supported the unidimensionality of the WEMBWS scale. Considering that IRT principles relate to the identification of most appropriate items for the evaluation of a specific level of a latent trait, items were evaluated and ranked in relation to their discrimination, difficulty, and reliability [26]. We considered various aspects of IRT including discrimination, difficulty, and information functions across thresholds of the latent trait and considering different levels. Specifically, most items yielded very high discriminative power apart from four items. The items that yielded high discrimination were, *“I’ve got energy to spare”*, *“I’ve been feeling cheerful”*, *“I’ve been able to make up my own mind about things”*, and *“I’ve been feeling loved”*. This shows that these four items were most distinguishable between high SWB and low SWB among gender. Specifically, clinicians should be more inclined to focus on items pertaining to having energy to spare, being cheerful, loved, and decisive to distinguish between those experiencing high and low levels of SWB among gender.

Further, while the level of difficulty of endorsing an item increased between the first (*“none of the time”*) and last options (*“all the time”*) of the Likert scale, the sequence of item difficulty varied across thresholds. Specifically, the ascending order of endorsed items between the first (*“none of the time”*) and second (*“rarely”*) options of the Likert scale was 1, 10, 8, 5, 14, 9, 13, 2, 6, 12, 7, 11, 4 and 3. However, the ascending order of endorsed items between the fourth (*“often”*) and last (*“all the time”*) options of the Likert scale was 1, 12, 8, 10, 11, 13, 7, 14, 9, 6, 2, 5, 4 and 3. This suggests that participants felt more inclined to endorse *“none of the time”* or *“rarely”* feeling optimistic about the future or feeling confident than feeling interested in other people and relaxed. Alternatively, participants felt more inclined to endorse *“often”* or *“always”* feeling optimistic about the future and feeling loved than feeling interested in other people and relaxed. Therefore, it is proposed that items should be interpreted differently when conducting clinical assessment of SWB.

Considering the scale (TIF), improved information performance was observed in the range between 2 SDs below and above the mean. However, considerable variation was observed in relation to the level of information precision provided by each criterion. More specifically, findings demonstrated that item 8 (*“I’ve been feeling good about myself”*) provided the highest level of information/reliability between 2 SD below and 1.5 SD above the mean. Items 14 (*“I’ve been feeling cheerful”*), 10 (*“I’ve been feeling confident”*), 1 (*“I’ve been feeling optimistic about the future”*) and 6 (*“I’ve been dealing with problems well”*) provided a considerable amount of information/reliability between 2 SDs below and above the mean. Finally, items 4 (*“I’ve been feeling interested in other people”*), 13 (*“I’ve been interested in new things”*), 12 (*“I’ve been feeling loved”*), and 11 (*“I’ve been able to make up my own mind about things”*) provided a consistently low amount of information/reliability between 3 SDs below and above the mean. However, these items along with item 2 (*“I’ve been feeling useful”*) and 7 (*“I’ve been thinking clearly”*) provided the most information between 2 and 3 SDs below the mean. This indicates that the following three-item sequence should be prioritised when attempting to identify participants with significantly low SWB: (i) *“I’ve been feeling interested in other people”*, (ii) *“I’ve been interested in new things”*, (iii) *“I’ve been feeling loved”*, (iv) *“I’ve been able to make up my own mind about things”*, (v) “*I’ve been able to make up my own mind about things”*, (vi) *“I’ve been feeling useful”*, and (vii) *“I’ve been thinking clearly”*. Lastly, the Test Characteristic Curve (TCC) demonstrated an appropriate steepness indicating that WEMWBS clearly identifies increments in SWB as the overall score increases. This favours WEMWBS as a sufficient psychometric measure for the assessment of individuals with high and low levels of SWB. Nonetheless, the instruments performance significantly decreases to differentiate very low (− 3 SD) and very high (+ 3 SD) SWB levels. Finally, considering the DIF analysis, results revealed that item 5 (*“I’ve got energy to spare”*) differed between men and women. The lack of scalar invariance in MCFA among genders regarding item 5 indicates that the same level of severity is not represented by the same responses across the two biological genders (i.e., a score of 2 for a female may not reflect the same severity as a score of 2 for a male). This, however, does not indicate the exact level of item difficulty applying for each of the two genders. The latter was addressed by IRT DIF, where it clearly indicated that men exhibited higher probability of endorsing ‘harder’ categories in item 5 (i.e., *“some of the time”*, *“often”*, and *“all of the time”*); suggesting that females experienced higher difficulty in addressing this item. This supports the MI analysis, where non-invariance as the intercept level between men and women differed for this item.

## Conclusion, limitations and further research

Firstly, we observed non-invariance for a single item, *“I’ve got energy to spare”*, which differed at the intercept level between men and women. Future research should explore whether this is a methodology issue with the psychometric questions or actual population differences between males and females. When this parameter was relaxed, the scale demonstrated MI, meaning all other items were valid at three levels between genders. Secondly, IRT analysis, using a GRM determined that the scale meets the assumptions fit to IRT analysis for discrimination and difficulty assessment. Following this, we found differing discriminative power across items with *“I’ve been feeling good about myself”*, *“I’ve been feeling cheerful”* and *“I’ve been feeling confident”* as having the strongest degree of discrimination. These items should be considered to differentially assess high and low levels of SWB than other items on the SWB scale. Item difficulty also indicated that the scale is most reliable at assessing SWB in non-clinical populations, but its reliable is decreases as scores deviate from the normative levels, particularly at clinically low levels. Future research utilising SWB scales should also consider psychological disorder diagnostics and exclude those meeting clinically significant criteria for psychological disorders relating to SWB. Alternatively, more discriminative items should be used to assess individuals with an extremely high or low state of SWB as outlined in this study. Results reported from this study provide information for clinicians and researchers to determine the appropriate use of the WEMBWS for their population of interest.

This analysis compliments existing research [36, 43, 53, 67], and is a worthwhile tool regarding increasing the quality of psychological questionnaires and psychological examination. Notwithstanding the unique innovative influence this study makes to the appraisal of WEMWBS psychometric properties, numerous limitations should be highlighted. Firstly, the employed sample included adult English speakers from developed countries and may lack a wide generalisability of application to samples involving non-English speakers, youth, and older adults. Secondly, considering that previous simulation studies observed low RMSEA rejection rates (< 0.001%) for samples *N* > 200 and *df* > 50, the proposed WEMWBS structure could be perhaps improved/revisited by a careful consideration of modification indices and/or estimation of correlated residuals [9]. Finally, IRT properties may not accurately reflect those experiencing pathological mental illness as a community sample of healthy adults was employed. Future studies may wish to address the shortcomings of the present study to improve and expand upon assessment practices typified by WEMWBS.

Conclusively, the present findings indicate that SWB evaluations and associations within gender based on WEMWBS should be interpreted with caution because of response pattern differences, which affect the metric and the scale properties of the instrument. Moreover, the instrument may not perform well for clinically low and high SWB levels and therefore, its use should be complemented with formal assessment (i.e., clinical interviews). Accordingly, as approximately one quarter of participants scored above 2 SD and were at risk for presenting SWB in the problematic range, further assessment should investigate these underlying causes or traits (i.e., obsessive compulsiveness; [14]) to provide more clarity on excessive levels of heightened SWB. Last, items differ considering their suitability to discriminate participants with different levels of the latent trait with certain items.

## Data Availability

The datasets used and/or analysed during the current study available from the corresponding author on reasonable request.
